# Epidemiology of influenza in pregnant women hospitalized with respiratory illness in Moscow, 2012/2013–2015/2016: a hospital-based active surveillance study

**DOI:** 10.1186/s12884-019-2192-z

**Published:** 2019-02-15

**Authors:** Svetlana Trushakova, Lidiya Kisteneva, Beatriz Guglieri-López, Evgenia Mukasheva, Irina Kruzhkova, Ainara Mira-Iglesias, Kirill Krasnoslobodtsev, Ekaterina Morozova, Ludmila Kolobukhina, Joan Puig-Barberà, Elena Burtseva

**Affiliations:** 1 Ministry of Health of the Russian Federation, FSBI “N.F. Gamaleya NRCEM”, 16, Gamaleya str, Moscow, Russia Moscow 123098 Russian Federation; 2 Fundación para el Fomento de la Investigación Sanitaria y Biomédica (FISABIO) de la Comunidad Valenciana, Avda Catalunya 21, 46020 Valencia, Spain

**Keywords:** Influenza, Pregnancy, Hospitalization, Surveillance

## Abstract

**Background:**

To better understand the impact of seasonal influenza in pregnant women we analyzed data collected during four seasons at a hospital for acute respiratory infection that specializes in treating pregnant women.

**Methods:**

This was a single-center active surveillance study of women 15–44 years of age hospitalized for acute respiratory diseases between 2012/2013 and 2015/2016 in Moscow, Russian Federation. Women had to have been hospitalized within 7 days of the onset of symptoms. Swabs were taken within 48 h of admission, and influenza was detected by reverse transcription-polymerase chain reaction.

**Results:**

During the four seasons, of the 1992 hospitalized women 1748 were pregnant. Laboratory-confirmed influenza was detected more frequently in pregnant women (825/1748; 47.2%) than non-pregnant women (58/244; 23.8%) (OR for influenza = 2.87 [95% CI, 2.10–3.92]; *p* <  0.001). This pattern was homogenous across seasons (*p* = 0.112 by test of homogeneity of equal odds). Influenza A(H1N1)pdm09 was the dominant strain in 2012/2013, A(H3N2) in 2013/2014, B/Yamagata lineage and A(H3N2) in 2014/2015, and A(H1N1)pdm09 in 2015/2016. Influenza-positive pregnant admissions went to the hospital sooner than influenza-negative pregnant admissions (*p* <  0.001). The risk of influenza increased by 2% with each year of age and was higher in women with underlying conditions (OR = 1.52 [95% CI, 1.16 to 1.99]). Pregnant women positive for influenza were homogeneously distributed by trimester (*p* = 0.37 for homogeneity; *p* = 0.49 for trend). Frequencies of stillbirth, delivery, preterm delivery, and caesarean delivery did not significantly differ between influenza-positive and influenza-negative hospitalized pregnant women or between subtypes/lineages.

**Conclusions:**

Pregnant women are at increased risk for hospitalization due to influenza irrespective of season, circulating viruses, or trimester.

**Electronic supplementary material:**

The online version of this article (10.1186/s12884-019-2192-z) contains supplementary material, which is available to authorized users.

## Background

Pregnant women are at increased risk of severe influenza illness and influenza-related death [1–3] and, during all trimesters, are at increased risk of hospital admission due to influenza infection [4]. Influenza illness during pregnancy also appears to be associated with increased rates of stillbirth, neonatal death, preterm delivery, and low birth weight [5–7]. In 2012, the World Health Organization expanded its recommendations for seasonal influenza vaccination to all pregnant women [8]. Maternal influenza vaccination does not pose a risk to the developing fetus [9] and may reduce stillbirth, growth restriction, and preterm birth [10–12].

Due to small study populations and designs that limit interpretation, the real impact of influenza on pregnant women remains uncertain [4, 13, 14]. Also, many of the severe cases of influenza analyzed occurred during the 2009 A(H1N1)pdm09 pandemic, when women may have been hospitalized for precautionary reasons [4]. Additional data are therefore needed to evaluate and support vaccination policies for pregnant women.

The present investigation was conducted as part of the Global Influenza Hospital Surveillance Network (GIHSN) that aims to generate data on the impact of influenza virus infection during hospitalization. The GIHSN is an international collaboration launched in 2012 to improve understanding of influenza epidemiology and better inform public health policy decisions [15]. The GIHSN has run a multinational, prospective, hospital-based active surveillance study to collect epidemiological data over several consecutive years. Sites included in the GIHSN use a standardized protocol and standard operating procedures, allowing results to be compared and pooled [16].

Since 2012, the Federal Budget Institute of Health “Clinical Hospital for Infectious Diseases No. 1” (CHID#1) in Moscow, Russian Federation has participated in the GIHSN. CHID#1 is one of two reference hospitals for acute respiratory diseases in Moscow and specializes in treating pregnant women. Here, we analyzed data collected from CHID#1 during the four seasons since its inclusion in the GIHSN (2012/2013 to 2015/2016) to describe the epidemiology of influenza in hospitalized pregnant women, and evaluate the clinical symptoms and outcomes of influenza-associated acute respiratory illness in this population.

## Methods

### Study design

This was a prospective, active surveillance hospital-based study conducted during the 2012/2013 to 2015/2016 influenza seasons at CHID#1 (Moscow, Russian Federation). CHID#1 is a unique, specialized department hospital for pregnant women with infectious diseases. The study was performed due to the study site’s participation in the GIHSN international influenza surveillance project and its use of the standardized GIHSN protocols [16].

### Study conduct

Patients admitted to the participating hospital were included, after written consent, if they were residents in the predefined hospital’s catchment area, presented with an acute illness possibly related to influenza, were not institutionalized, and were admitted within 7 days of the onset of symptoms. Patients discharged during the previous 30 days were excluded. Swabs were collected within 48 h from patients meeting the inclusion criteria and tested by reverse transcription-polymerase chain reaction (RT-PCR) for influenza. Influenza-positive samples were sub-typed by RT-PCR to identify A(H1N1)pdm09, A(H3N2), B/Yamagata-lineage, and B/Victoria-lineage strains.

The present analysis was limited to women 15–44 years of age admitted with an acute respiratory infection, in line with the age range used by others [3, 17, 18]. All other aspects of patient selection were in accordance with the GIHSN study protocol [16]. RT-PCR was conducted at the World Health Organization National Influenza Centre at the Ivanovsky Institute of Virology (Moscow, Russian Federation) using Amplisens® Ribo-sorb and Ribo-prep (Federal Budget Institute of Science “Central Research Institute for Epidemiology”, Moscow, Russian Federation) or a PREP-NA DNA/RNA extraction kit (DNA-Technology, Moscow, Russian Federation) to extract RNA; a Reverta-L kit (Federal Budget Institute of Science “Central Research Institute for Epidemiology”) for reverse transcription; and kits from Federal Budget Institute of Science “Central Research Institute for Epidemiology” and DNA-Technology to amplify influenza A, A(H1N1), A(H3N2), A(H1N1)pdm09, and B genes.

Socio-demographic and clinical information were collected by face-to-face interviews with patients or attending physicians or by reviewing clinical records. Information collected included socio-demographic characteristics, the major complaint at admission, smoking habits, underlying conditions, vaccination status, pregnancy outcome during the current admission, clinical course, and major diagnosis at discharge. Registered pregnancy outcomes included abortion (terminated at < 20 weeks gestational age), stillbirth (≥ 20 weeks gestational age with no heartbeat or respiratory effort), delivery (birth at any gestational age with heartbeat or respiratory effort), live preterm birth (< 37 weeks of gestation), low birth weight (< 2500 g), perinatal death (i.e. during the mother’s current admission), and caesarean delivery. Main discharge diagnoses were recorded by the physician using ICD-10 codes.

### Statistical analysis

Statistical analyses were restricted to women recruited in periods with continuous influenza circulation, defined as ≥2 admissions positive for influenza in ≥2 consecutive weeks. Calendar time (admission week) was modeled using restricted cubic splines with four knots. The number of knots was set based on the Akaike information criterion [19]. The odds ratio (OR) of admission with influenza was calculated by bivariate logistic regression using the category with the lowest value as the reference. To estimate the significance of differences among groups, chi-squared, likelihood ratio, t, and nonparametric K-sample tests were used. For comparisons of continuous variables among multiple categories, equality of medians and one-way analysis of variance were used with Scheffe correction for multiple comparisons. Likelihood ratio tests were used to check for confounding, interaction, linearity, and clustering. The adjusted odds ratio (aOR) of influenza among pregnant women was estimated by multivariate logistic regression using minimal sufficient adjustment by variables identified as confounders by causal diagrams (e.g. age, underlying conditions, smoking habits, admissions in the previous year, time to swab, season, and epidemiological week at admission). The goodness of fit of the models was assessed using Akaike and Bayesian information criteria. Conditional plots [20] of the average predicted probability of influenza positive admission and of pregnancy outcome during admission were used to assess complex relationships between the pregnant women’s age in years, their infant’s gestational age, underlying conditions, influenza infection, and infection by A subtype or B lineage. The predicted probabilities of either influenza infection or pregnancy outcomes during admission were adjusted by age, smoking habits, chronic underlying conditions, admission in previous 12 months, pregnancy trimester, time to swab, and calendar time (season-week) as restricted cubic splines. All *p*-values were two-tailed. A *p*-value < 0.05 was considered to indicate statistical significance. Heterogeneity in the effects of risk factors were quantified using the I^2^ test, with heterogeneity defined as an I^2^ > 50%. All statistical analyses were performed with Stata/SE version 14 (College Station, TX, USA).

## Results

### Included population

The study included 1992 women 15–44 years old admitted for acute respiratory diseases between 2012/2013 and 2015/2016. Of these admissions, 1748 were pregnant (Table 1). Influenza was detected in 47.2% (825/1748) of pregnant admissions and 23.8% (58/244) of non-pregnant admissions (OR for influenza = 2.87 [95% confidence interval (CI), 2.10–3.92]; *p* <  0.001; data not shown). Proportions of pregnant admissions with influenza were similar during the four influenza seasons included (48.7% in 2012/2013, 44.5% in 2013/2014, 52.4% in 2014/2015, and 44.9% in 2015/2016; *p* = 0.112 by test for homogeneity of equal odds; data not shown). Proportions of non-pregnant women with influenza were not analyzed further because of insufficient numbers. The main comparative assessment and conclusions were made by comparing hospitalized influenza-positive pregnant women with hospitalized influenza-negative pregnant women.

**Table 1:** Influenza infection status in pregnant admissions

	n (%)				
	2012/2013	2013/2014	2014/2015	2015/2016	All seasons
RT-PCR result	*N* = 520	*N* = 335	*N* = 296	*N* = 597	*N* = 1748
RT-PCR result					
Positive^a^	253 (48.7)	149 (44.5)	155 (52.4)	268 (44.9)	825 (47.2)
Negative	267 (51.3)	186 (55.5)	141 (47.6)	329 (55.1)	923 (52.8)
Influenza type					
A(H1N1)pdm09	155 (61.3)	13 (8.7)	11 (7.1)	182 (67.9)	361 (43.8)
A(H3N2)	41 (16.2)	100 (67.1)	62 (40.0)	21 (7.8)	224 (27.2)
B/Yamagata lineage	6 (2.4)	34 (22.8)	66 (42.6)	0 (0.0)	106 (12.8)
B/Victoria lineage	10 (4.0)	2 (1.3)	5 (3.2)	59 (22.0)	76 (9.2)
A not subtyped	5 (2.0)	0 (0.0)	1 (0.6)	2 (0.7)	8 (1.0)
B undetermined lineage	36 (14.2)	0 (0.0)	10 (6.5)	4 (1.5)	50 (6.1)

Abbreviation: *RT-PCR* reverse transcription-polymerase chain reaction

^a^Test of homogeneity (equal odds) between seasons: *p* = 0.1176

### Influenza circulation

**Fig. 1:**
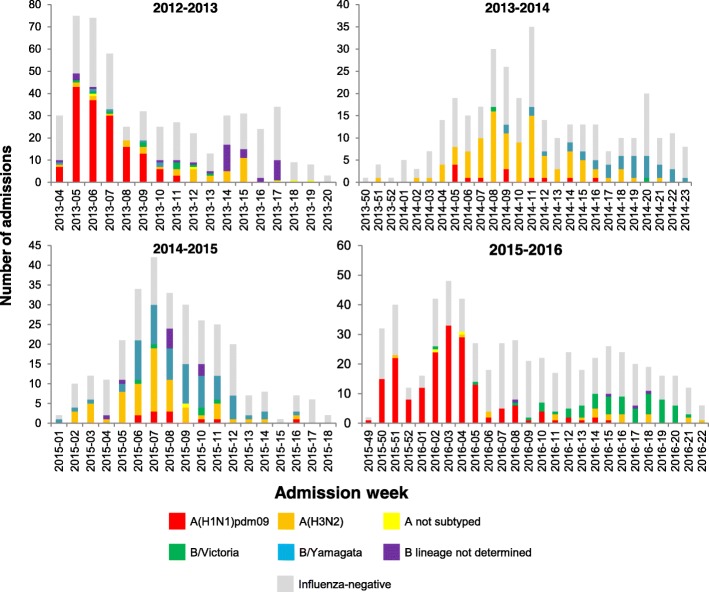
Number of pregnant admissions with acute respiratory infection by influenza subtype/lineage and season

During the four influenza seasons included in this study, influenza was detected during similar periods, although the season varied from as short as 13 weeks in 2014/2015 to as long as 24 weeks in 2015/2016, and the peak occurred as early as week 3 in 2015/2016 and as late as week 11 in 2013/2014 (Fig. 1). Influenza A(H1N1)pdm09 was the dominant strain in 2012/2013, A(H3N2) in 2013/2014, B/Yamagata lineage and A(H3N2) in 2014/2015, and A(H1N1)pdm09 in 2015/2016 (Table 1 and Fig. 1).

### Characteristics of influenza-positive pregnant admissions

Influenza-positive pregnant admissions were slightly older than influenza-negative pregnant admissions (median = 28.5 vs. 28.0 years; OR = 1.02 [95% CI, 1.00–1.04]; *p* = 0.021) (Table 2). The risk of a positive influenza result increased with each year by 2% (95% CI, 0–4%). When analyzed by subtype/lineage, the probability of influenza infection increased with each additional year by 3% (95% CI, 1–6%; *p* = 0.012) for A(H1N1)pdm09 and by 9% (4–13%; *p* <  0.001) for B/Yamagata lineage but was unaffected by age for A(H3N2) and B/Victoria lineage (Table 4). Median ages were higher for pregnant admissions positive for B/Yamagata-lineage viruses (30 years) than for those positive for A(H1N1)pdm09 (29 years; *p* = 0.01), A(H3N2) (28 years; *p* = 0.006), or B/Victoria lineage (28 years; *p* = 0.006) (Table 3). Minor but significant differences in age were found for pregnant admissions by influenza subtype/lineage (*p* = 0.028).

**Table 2:** Characteristics of pregnant admissions by influenza infection status

Characteristic	Influenza positive	Influenza negative	OR (95% CI)	*P*-value
	*N* = 825	N = 923		
Age group, n (%)				
15–19 years	27 (3.3)	42 (4.6)	1	
20–24 years	153 (18.6)	214 (23.2)	1.11 (0.66–1.88)	0.692
25–29 years	327 (39.6)	341 (36.9)	1.49 (0.90–2.48)	0.122
30–34 years	206 (25.0)	214 (23.2)	1.50 (0.89–2.52)	0.128
35–39 years	89 (10.8)	94 (10.2)	1.47 (0.84–2.59)	0.178
40–44 years	23 (2.8)	18 (2.0)	1.99 (0.91–4.35)	0.086
Median age in years (range)	28.5 (15–44)	28 (15–44)	1.02 (1.00–1.04)	0.021
Underlying conditions, n (%)				
One or more	136 (16.5)	106 (11.5)	1.52 (1.16–1.99)	0.003
Cardiovascular disease	28 (3.4)	23 (2.5)	1.37 (0.78–2.40)	0.268
Chronic obstructive pulmonary disease	12 (1.5)	8 (0.9)	1.68 (0.69–4.14)	0.256
Asthma	10 (1.2)	6 (0.7)	1.87 (0.68–5.17)	0.227
Diabetes ^a^	0 (0.0)	3 (0.3)	0.29 (0.00–2.71)	0.294
Renal impairment	68 (8.2)	54 (5.9)	1.44 (0.99–2.09)	0.053
Cirrhosis	9 (1.1)	8 (0.9)	1.26 (0.48–3.28)	0.637
Neuromuscular ^a^	1 (0.1)	0 (0.0)	1.12 (0.03–∞)	0.944
Neoplasm	2 (0.2)	2 (0.2)	1.12 (0.16–7.95)	0.912
Rheumatic disease ^a^	0 (0.0)	1 (0.1)	1.12 (0.00–43.63)	1.000
Antiviral treatment before admission, n (%)	8 (1.0)	11 (1.2)	0.81 (0.32–2.02)	0.652
Influenza vaccination, n (%)	3 (0.4)	3 (0.3)	0.99 (0.97–1.01)	0.392
Trimester of pregnancy, n (%)				
First (0–13 weeks)	177 (21.5)	221 (23.9)	1	
Second (14–26 weeks)	355 (43.0)	372 (40.3)	1.19 (0.93–1.52)	0.162
Third (27–42 weeks)	291 (35.3)	325 (35.2)	1.12 (0.87–1.44)	0.388
Gestational age at admission (weeks), median (range)	23 (1–41) ^b^	23 (3–42) ^c^	1.00 (0.99–1.01)	0.872
Smoking habits, n (%)				
Never smoked	563 (68.2)	602 (65.2)	1	
Past smoker	225 (27.3)	268 (29.0)	0.90 (0.73–1.11)	0.317
Current smoker	37 (4.5)	53 (5.7)	0.75 (0.48–1.15)	0.188
Socioeconomic class, n (%)				
Managerial	359 (43.5)	383 (41.5)	1	
Skilled	360 (43.6)	425 (46.0)	0.9 (0.74–1.10)	0.324
Unskilled	34 (4.1)	52 (5.6)	0.7 (0.44–1.10)	0.121
Other	72 (8.7)	63 (6.8)	1.22 (0.84–1.76)	0.29
Time from onset of symptoms to swabbing, n (%)				
0–2 days	559 (67.8)	541 (58.6)	1	
3–4 days	228 (27.6)	280 (30.3)	0.79 (0.64–0.97)	0.027
5–7 days	38 (4.6)	102 (11.1)	0.36 (0.24–0.53)	0.001
Days from onset of symptoms to swabbing, median (range)	2 (0–7)	2 (0–7)	0.87 (0.82–0.93)	0.001

^a^Odds ratio estimated by exact logistic regression

^b^*N* = 823

^c^*N* = 918

**Table 3:** Characteristics of pregnant admissions by influenza subtype/lineage

Characteristic	A(H1N1)pdm09	A(H3N2)	B/Yamagata	B/Victoria	*P*-value
	*N* = 361	*N* = 224	*N* = 106	*N* = 76	
Age group, n (%)					0.028
15–19 years	11 (3.0)	10 (4.5)	2 (1.9)	2 (2.6)	
20–24 years	69 (19.1)	40 (17.9)	17 (16.0)	19 (25.0)	
25–29 years	146 (40.4)	95 (42.4)	31 (29.2)	30 (39.5)	
30–34 years	91 (25.2)	51 (22.8)	31 (29.2)	23 (30.3)	
35–39 years	37 (10.2)	21 (9.4)	18 (17.0)	1 (1.3)	
40–44 years	7 (1.9)	7 (3.1)	7 (6.6)	1 (1.3)	
Age (years), median (range)	29 (17–44)	28 (15–43)	30 (19–42)	28 (19–45)	0.004
Underlying conditions ^a^, n (%)					
None	300 (83.1)	182 (81.3)	92 (86.8)	63 (82.9)	0.666
One or more	61 (16.9)	42 (18.8)	14 (13.2)	13 (17.1)	0.666
Cardiovascular disease	16 (4.4)	7 (3.1)	1 (0.9)	3 (3.9)	0.376
Chronic obstructive pulmonary disease	7 (1.9)	5 (2.2)	0 (0.0)	0 (0.0)	0.276
Asthma	4 (1.1)	3 (1.3)	1 (0.9)	1 (1.3)	0.988
Renal impairment	31 (8.6)	20 (8.9)	7 (6.6)	8 (10.5)	0.819
Cirrhosis	3 (0.8)	2 (0.9)	2 (1.9)	1 (1.3)	0.803
Neoplasm	2 (0.6)	0 (0.0)	0 (0.0)	0 (0.0)	0.521
Trimester of pregnancy, n (%)					0.005
First (0–13 weeks)	87 (24.1)	46 (20.5)	17 (16.0)	11 (14.5)	
Second (14–26 weeks)	167 (46.3)	81 (36.2)	44 (41.5)	40 (52.6)	
Third (27–42 weeks)	106 (29.4)	97 (43.3)	45 (42.5)	25 (32.9)	
Gestational age at admission (weeks), median (range)	21 (2–41)	25 (4–40)	24 (7–41)	27 (7–38)	0.017
Time from onset of symptoms to swabbing, n (%)					
0–2 days	263 (72.9)	167 (74.6)	57 (53.8)	42 (55.3)	0.001
3–4 days	83 (23.0)	48 (21.4)	47 (44.3)	29 (38.2)	
5–7 days	15 (4.2)	9 (4.0)	2 (1.9)	5 (6.6)	
Days from onset of symptoms to swabbing, median (range)	2 (0–7)	2 (0–6)	2 (0–5)	2 (0–6)	0.001

Abbreviation: *NC* not calculated

^a^Diabetes, neuromuscular disease, and rheumatic disease are not listed because of low numbers

Pregnant admissions who were positive for influenza more frequently had underlying conditions than those who were negative for influenza (OR = 1.52 [95% CI, 1.16–1.99], *p* = 0.003; aOR = 1.56 [95% CI, 1.16–2.08]) (Tables 2 and 4). This was confirmed for influenza A(H3N2) and A(H1N1) but not for the two B lineages (Table 4). However, significant differences between influenza-positive and influenza-negative pregnant admissions were not detected for the individual underlying conditions (Tables 2 and 3).

**Table 4:** Adjusted estimates of risk factors for influenza in pregnant admissions overall and by subtype/lineage

	Adjusted odds ratio (95% CI) ^a^				
	Any influenza	A(H1N1)pdm09	A(H3N2)	B/Yamagata	B/Victoria
Characteristic	N = 825	N = 361	N = 224	N = 106	N = 76
Age (in years) ^b^	1.02 (1.00–1.04)	1.03 (1.01–1.06)	1.00 (0.97–1.03)	1.09 (1.04–1.13)	0.96 (0.91–1.00)
Trimester					
First (0–13 weeks)	1	1	1	1	1
Second (14–26 weeks)	1.19 (0.92–1.53)	1.17 (0.82–1.66)	0.89 (0.58–1.35)	1.34 (0.75–2.51)	2.28 (1.10–4.74)
Third (27–42 weeks)	1.12 (0.86–1.46)	0.82 (0.56–1.19)	1.23 (0.82–1.87)	1.57 (0.86–2.88)	1.58 (0.72–3.44)
Underlying conditions					
No	1	1	1	1	1
Yes	1.56 (1.16–2.08)	1.78 (1.19–2.67)	1.91 (1.25–2.93)	1.24 (0.66–2.34)	2.01 (0.39–3.15)

^a^Adjusted for age, trimester, comorbidity, hospital admission in the previous 12 months, time to swab, and season-week

^b^Comparison group was influenza-negative pregnant women

Gestational age distribution was similar in influenza-positive and influenza-negative pregnant admissions (*p* = 0.872) (Table 2). Pregnant admissions positive for influenza were homogeneously distributed by trimester (*p* = 0.37 for homogeneity in the distribution of estimates and *p* = 0.49 for trend, data not shown). The OR of admission with any influenza did not differ between the first and second trimesters (1.19 [95% CI, 0.92–1.53]; *p* = 0.16) or between the first and third trimesters (1.12 [95% CI, 0.86–1.46]; *p* = 0.39). Most of the pregnant admissions infected with influenza A(H1N1)pdm09 or B/Victoria lineage were in the first or second trimester, whereas most of those infected with influenza A(H3N2) or B/Yamagata lineage were in the second or third trimester, resulting in a significant difference in strain distribution by trimester (*p* = 0.005) (Table 3); however, aORs for each strain did not differ between trimesters (Table 4). Likewise, distributions of gestational age at admission differed significantly by virus subtype/lineage, and median gestational ages were lower for admissions positive for A(H1N1)pdm09 (21 weeks) than for admissions positive for A(H3N2) (25 weeks; *p* = 0.032 [data not shown]) or B/Yamagata lineage (24 weeks; *p* = 0.027 [data not shown]) (Table 3). Conditional plots did not reveal interactions between trimester and patient age or presence of underlying conditions for the risk of admission with any influenza or with each subtype/lineage (Additional file 1).

Rates of influenza vaccination and antiviral use before admission were ≤ 1% and did not differ between influenza-positive and -negative pregnant admissions (Table 2). Smoking habits and socioeconomic class also did not differ between pregnant admissions positive or negative for influenza.

The probability of laboratory-confirmed influenza decreased with the time between symptom onset and swabbing (Table 2). The probability was lowest in pregnant admissions with samples taken 5–7 days after the onset of symptoms (OR = 0.36 [95% CI, 0.24–0.53]; *p* = 0.001). The adjusted probability of a positive result decreased 13% (95% CI, 7–18%) for each day between onset of symptoms and swabbing (aOR = 0.87 [95% CI, 0.82–0.93]). Time to swabbing differed significantly between subtypes/lineages, although the median was 2 days in all cases (Table 3).

### Clinical manifestations of influenza in pregnant admissions and pregnancy outcomes

Fever (*p* <  0.001), cough (*p* <  0.001), and myalgia (*p* <  0.001) were reported as presenting complaints more often in influenza-positive than influenza-negative pregnant admissions (Table 5). Overall, fever, reported by 97.1%, was the most common presenting complaint in pregnant admissions positive for influenza. The aOR for influenza-positive vs. influenza-negative pregnant admissions was 6.34 (95% CI, 4.01–10.03) for fever and 2.76 (95% CI, 2.13–3.43) for cough (Table 6). Cough was a common presenting complaint in pregnant admissions infected with B/Victoria lineage (86.8%) and A(H1N1)pdm09 (82.0%) but less common for those infected with A(H3N2) (69.2%) or B/Yamagata lineage (72.6%) (*p* <  0.001) (Table 7). Proportions of pregnant admissions reporting all other symptoms (headache, malaise, myalgia, sore throat, and dyspnea) also differed significantly by subtype/lineage. For example, dyspnea was a major presenting complaint in 18.0% of admissions with A(H1N1)pdm09 but in less than 5% of admissions with A(H3N2) or B/Yamagata-lineage and in 6.6of admissions with B/Victoria-lineage.

**Table 5:** Clinical manifestations and outcomes in pregnant admissions by influenza infection status

	Influenza positive	Influenza negative	Odds ratio (95% CI) ^a^	
Clinical manifestation/outcome	N = 825	N = 923		*P*-value
Signs/symptoms, n (%)				
Fever	801 (97.1)	781 (84.6)	6.07 (3.89–9.46)	< 0.001
Headache	445 (53.9)	489 (53.0)	1.04 (0.86–1.25)	0.688
Malaise	497 (60.2)	550 (59.6)	1.03 (0.85–1.24)	0.781
Myalgia	354 (42.9)	244 (26.4)	2.09 (1.71–2.56)	< 0.001
Cough	630 (76.4)	511 (55.4)	2.60 (2.12–3.20)	< 0.001
Sore throat	546 (66.2)	689 (74.6)	0.66 (0.54–0.82)	< 0.001
Dyspnea	86 (10.4)	75 (8.1)	1.32 (0.95–1.82)	0.098
Time from onset of symptoms to admission, n (%)				
1 day	392 (47.5)	384 (41.6)	1	
2 days	209 (25.3)	194 (21.0)	1.06 (0.83–1.34)	0.661
3 days	138 (16.7)	158 (17.1)	0.86 (0.65–1.12)	0.254
≥ 4 days	86 (10.4)	187 (20.3)	0.45 (0.34–0.60)	< 0.001
Time from onset of symptoms to admission, median (range)	2 (0–6)	2 (0–7)	0.85 (0.80–0.91)	< 0.001
Length of hospital stay, n (%) ^b^				
0–4 days	254 (30.8)	342 (37.1)	1	
5 days	155 (18.8)	128 (13.9)	1.63 (1.23–2.17)	< 0.001
6–7 days	261 (31.6)	259 (28.1)	1.36 (1.07–1.72)	< 0.001
Median length of hospital stay (range)	6 (0–35)	5 (0–31)	1.00 (0.98–1.04)	0.627
Intensive care unit admission, n (%)	1 (0.1)	2 (0.2)	0.56 (0.05–6.17)	0.635
Pregnancy outcome, n (%)				
Aborted (< 20 weeks)	14 (1.7)	39 (4.2)	0.39 (0.21–0.73)	0.003
Stillborn (≥ 20 weeks)	12 (1.5)	11 (1.2)	1.22 (0.54–2.79)	0.631
Live delivery during the current admission, n (%)	78 (9.5)	99 (10.7)	0.87 (0.64–1.19)	0.379
Preterm (< 37 weeks) ^c^	17 (21.8)	19 (19.2)	1.17 (0.56–2.45)	0.669
Cesarean‡	14 (17.9)	16 (16.2)	1.13 (0.52–2.50)	0.753
Low birth weight (< 2500 g) ^c^	3 (3.8)	12 (12.1)	0.29 (0.08–1.07)	0.062
Discharge diagnosis, n (%)				
Influenza	617 (74.8)	108 (11.7)	23.34 (18.07–30.13)	< 0.001
Pneumonia	9 (1.1)	13 (1.4)	0.77 (0.33–1.82)	0.553
Respiratory disease	187 (22.7)	736 (79.7)	0.74 (0.06–0.09)	< 0.001
Other	12 (1.5)	66 (7.2)	0.19 (0.10–0.36)	< 0.001

^a^Estimated by exact logistic regression

^b^Proportions are relative to deliveries during the current admission

N = 825 for influenza positive, *N* = 922 for influenza negative

**Table 6:** Adjusted estimates of risks for clinical manifestations and outcomes in pregnant admissions overall and by subtype/lineage

	Adjusted odds ratio (95% CI) ^a^				
	Any influenza	A(H1N1)pdm09	A(H3N2)	B/Yamagata	B/Victoria
Characteristic	N = 825	N = 361	N = 224	N = 106	N = 76
Signs and symptoms					
Fever	6.34 (4.01–10.03)	5.81 (3.02–11.17)	6.36 (2.88–10.65)	11.0 (2.65–45.73)	5.56 (1.31–23.51)
Headache	1.03 (0.84–1.25)	1.35 (1.02–1.77)	0.84 (0.62–1.14)	0.72 (0.47–1.09)	1.41 (0.83–2.40)
Malaise	1.05 (0.86–1.29)	1.67 (1.26–2.22)	0.72 (0.53–0.98)	0.56 (0.37–0.85)	1.84 (1.01–3.33)
Myalgia	1.83 (1.48–2.26)	2.62 (1.98–3.47)	1.16 (0.83–1.62)	1.71 (1.11–2.63)	2.12 (1.28–3.52)
Cough	2.76 (2.13–3.43)	4.21 (3.04–5.83)	2.03 (1.47–2.82)	2.14 (1.35–3.38)	4.72 (2.35–9.45)
Sore throat	0.64 (0.51–0.80)	0.53 (0.39–0.71)	0.71 (0.50–1.00)	0.57 (0.36–0.89)	0.78 (0.44–1.39)
Dyspnea	1.10 (0.78–1.54)	2.31 (1.54–3.49)	0.37 (0.18–0.77)	0.42 (0.16–1.08)	0.98 (0.37–2.63)
Days between symptom onset and admission					
Below median	1	1	1	1	1
Median or above	1.43 (0.88–2.32)	2.06 (1.12–3.79)	1.29 (0.56–2.99)	1.15 (0.43–3.13)	0.96 (0.26–3.51)
Length of stay					
Below median	1	1	1	1	1
Median or above	0.94 (0.77–1.14)	1.34 (1.02–176)	0.72 (0.53–0.98)	0.52 (0.34–0.80)	1.08 (0.65–1.77)
Pregnancy outcome					
Abortion	0.32 (0.16–0.64)	0.44 (0.18–1.09)	0.30 (0.09–1.05)	0.36 (0.80–1.67)	–
Stillbirth	1.10 (0.47–2.59)	0.35 (0.07–1.72)	1.14 (0.30–4.35)	3 (0.87–10.28)	1.34 (0.25–7.06)
Live delivery	0.74 (0.52–1.06)	0.78 (0.47–1.29)	0.64 (0.37–1.10)	0.90 (0.45–1.78)	0.62 (0.20–1.94)
Preterm delivery	0.89 (0.45–1.78)	1.07 (0.45–2.56)	0.67 (0.22–2.06)	0.42 (0.05–3.31)	2.42 (0.47–2.38)
Cesarean delivery	0.96 (0.45–2.06)	0.46 (0.12–1.70)	1.22 (0.44–3.42)	0.87 (0.18–4.07)	–
Low birth weight	0.34 (0.10–1.10)	0.28 (0.03–2.13)	0.42 (0.09–2.06)	–	2.85 (0.29–1.07)

^a^Adjusted for age, trimester, comorbidity, hospital admission in the previous 12 months, time to swab, and season-week

**Table 7:** Clinical manifestations and outcomes in pregnant admissions by influenza subtype/lineage

	A(H1N1)pdm09	A(H3N2)	B/Yamagata	B/Victoria	
Manifestation/outcome	N = 361	N = 224	N = 106	N = 76	*P*-value
Signs/symptoms, n (%)					
Fever	350 (97.0)	217 (96.9)	104 (98.1)	74 (97.4)	0.924
Headache	209 (57.9)	110 (49.1)	48 (45.3)	52 (68.4)	0.003
Malaise	240 (66.5)	114 (50.9)	51 (48.1)	60 (78.9)	< 0.001
Myalgia	191 (52.9)	73 (32.6)	42 (39.6)	35 (46.1)	< 0.001
Cough	296 (82.0)	155 (69.2)	77 (72.6)	66 (86.8)	< 0.001
Sore throat	215 (59.6)	160 (71.4)	71 (67.0)	56 (73.7)	0.009
Dyspnea	65 (18.0)	9 (4.0)	5 (4.7)	5 (6.6)	< 0.001
Time from onset of symptoms to admission, n (%)					< 0.001
1 day	195 (54.0)	115 (51.3)	29 (27.4)	31 (40.8)	
2 days	98 (27.1)	58 (25.9)	32 (30.2)	13 (17.1)	
3 days	40 (11.1)	31 (13.8)	35 (33.0)	16 (21.1)	
≥ 4 days	28 (7.8)	20 (8.9)	10 (9.4)	16 (21.1)	
Time from onset of symptoms to admission (days), median (range)	1 (0–6)	1 (0–6)	2 (0–5)	2 (0–5)	< 0.001
Length of hospitalization stay, n (%) ^a^					< 0.001
0–4 days	124 (34.3)	61 (27.2)	22 (20.8)	26 (34.2)	
5 days	85 (23.5)	32 (14.3)	14 (13.2)	16 (21.1)	
6–7 days	100 (27.7)	84 (37.5)	39 (36.8)	26 (34.2)	
≥ 8 days	52 (14.4)	46 (20.5)	30 (28.3)	8 (10.5)	
Length of hospitalization stay (days), median (range)	5 (0–35)	6 (0–14)	6 (1–16)	5 (0–11)	< 0.001
Admission to an intensive care unit, n (%)	1 (0.3)	0 (0.0)	0 (0.0)	0 (0.0)	0.771
Pregnancy outcome, n (%)					
Abortion (< 20 weeks)	8 (2.2)	3 (1.3)	2 (1.9)	0 (0.0)	0.667
Stillbirth (≥ 20 weeks)	2 (0.6)	3 (12.5)	4 (3.8)	2 (2.6)	0.050
Live delivery during the current admission	31 (8.6)	24 (10.7)	14 (13.2)	4 (5.3)	0.261
Preterm (< 37 weeks gestational age) ^b^	9 (29.0)	4 (16.7)	1 (7.1)	2 (50.0)	0.177
Cesarean†	3 (9.7)	6 (25.0)	2 (14.3)	0 (0.0)	0.391
Low birth weight (< 2500 g) ^c^	1 (3.2)	1 (4.2)	0 (0.0)	1 (25.0)	0.276

^a^*N* = 223 for A(H3N2) and *N* = 105 for B/Yamagata

^b^Proportions are relative to deliveries

Hospital admission occurred sooner after the onset of symptoms in influenza-positive than influenza-negative pregnant admissions (*p* <  0.001) (Table 5). This was particularly the case for pregnant admissions positive for influenza A(H1N1)pdm09 and influenza A(H3N2), where more than half went to the hospital on the first day (Table 7). In contrast, more than half of pregnant admissions positive for influenza B went to the hospital by the second day. As a result, the time to symptom onset differed significantly by strain (*p* <  0.001).

The overall length of hospital stay was not significantly longer for pregnant admissions positive for influenza than those negative for influenza (Table 5); however, pregnant women hospitalized for > 4 days had a higher probability of laboratory-confirmed influenza than those hospitalized for ≤4 days (OR = 1.62 [95%CI, 1.21–2.16]; *p* = 0.001; data not shown). The median hospital stay also differed by subtype/lineage (Table 7) and was longer for pregnant women infected with B/Yamagata lineage (6.4 days) than for those infected with influenza B/Victoria lineage (4.9 days; *p* = 0.025 [data not shown]) or A(H1N1)pdm09 (5.4 days; *p* = 0.074 [data not shown]) but did not differ for those infected with A(H3N2) (5.9 days; *p* > 0.05 [data not shown]).

At discharge, the most common diagnosis was other respiratory infections (*n* = 923; 52.8%) followed by influenza (*n* = 725; 41.5%) (Table 5). Pneumonia was the main discharge diagnosis in 22 cases (0.1%) and did not differ between influenza-positive and influenza-negative pregnant women.

Only one influenza-positive pregnant admission and only two influenza-negative pregnant admissions were hospitalized in an intensive care unit (Table 5). No deaths were reported.

### Pregnancy outcomes

Pregnancy outcomes recorded during the hospital stay included 177 live births, 53 abortions (pregnancy stopped before 20 weeks), and 23 stillbirths (Table 5). No perinatal deaths were reported (data not shown).

**Fig. 2:**
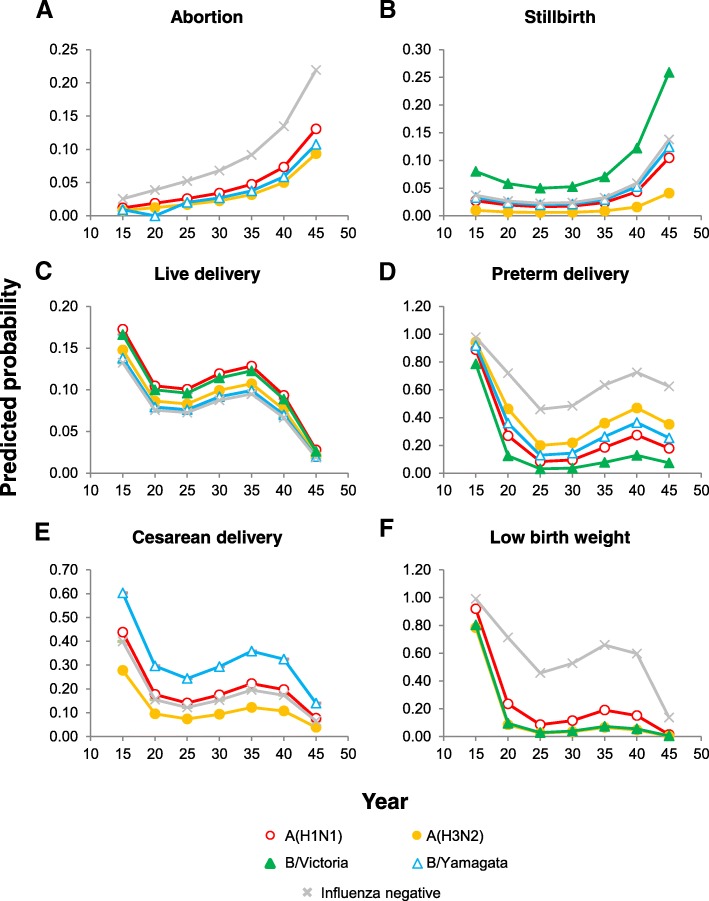
Predicted probabilities of birth outcomes according to the mother’s age and influenza strain. Predicted probabilities of abortion (**a**), stillbirth (**b**), live delivery (**c**), preterm delivery **(d**), cesarean delivery (**e**), and low birth weight (**f**) in influenza-positive pregnant admissions. Probabilities were adjusted by age, smoking habits, chronic underlying conditions, previous admissions, trimester, time to swab, and season-week

Abortion was more frequent in influenza-negative than influenza-positive pregnant admissions (4.2% vs. 1.7%; *p* = 0.003), although the frequency did not significantly differ by subtype/lineage (Table 7). Frequencies of other pregnancy outcomes did not differ between strains, but predicted probabilities were higher for stillbirth in women infected with B/Yamagata, for cesarean delivery in women infected with A(H3N2), and for preterm delivery and low birth weight in women infected with B/Victoria (Table 8 and Fig. 2).

**Table 8:** Predicted probability of pregnancy outcomes during admission according to influenza infection status

Outcome	RT-PCR result	Adjusted predicted probability ^a^ (95% CI)	n
Abortion (< 20 weeks)	No influenza	0.07 (0.05, 0.09)	39
	A(H1N1)pdm09	0.03 (0.01, 0.06)	8
	A(H3N2)	0.02 (0.00, 0.05)	3
	B/Yamagata-lineage	0.03 (−0.01, 0.06)	2
Stillbirth (≥ 20 weeks)	No influenza	0.02 (0.01, 0.03)	11
	A(H1N1)pdm09	0.01 (0.00, 0.02)	2
	A(H3N2)	0.02 (0.00, 0.05)	3
	B/Yamagata-lineage	0.06 (0.00, 0.12)	4
	B/Victoria-lineage	0.03 (−0.01, 0.07)	2
Live delivery during admission	No influenza	0.11 (0.09, 0.13)	99
	A(H1N1)pdm09	0.09 (0.06, 0.12)	31
	A(H3N2)	0.09 (0.05, 0.12)	24
	B/Yamagata-lineage	0.11 (0.06, 0.16)	14
	B/Victoria-lineage	0.08 (0.01, 0.15)	4
Preterm (< 37 weeks gestational age)	No influenza	0.13 (0.07, 0.20)	19
	A(H1N1)pdm09	0.27 (0.06, 0.48)	9
	A(H3N2)	0.19 (0.01, 0.38)	4
	B/Yamagata-lineage	0.06 (−0.05, 0.17)	1
	B/Victoria-lineage	0.54 (− 0.05, 1.13)	2
Cesarean delivery	No influenza	0.18 (0.09, 0.26)	16
	A(H1N1)pdm09	0.09 (−0.02, 0.21)	3
	A(H3N2)	0.29 (0.09, 0.49)	6
	B/Yamagata-lineage	0.15 (−0.05, 0.36)	2
Low birth weight (< 2500 g)	No influenza	0.14 (0.07, 0.21)	12
	A(H1N1)pdm09	0.05 (−0.06, 0.16)	1
	A(H3N2)	0.06 (−0.06, 0.17)	1
	B/Victoria-lineage	0.55 (−0.05, 1.15)	1

Abbreviation: *RT-PCR* reverse transcription-polymerase chain reaction

^a^Adjusted by age, smoking habits, chronic underlying conditions, admission in last 12 months, trimester, time to swab and season-week

Age had a noticeable impact on pregnancy outcomes in influenza-positive admissions. The probabilities of abortion and stillbirth increased with the mother’s age, whereas the probability of live birth decreased with the mother’s age (Fig. 2). Probabilities of preterm delivery, caesarean delivery, and low birth weight were highest in the youngest mothers, although a second smaller increase in probability occurred in women 35–40 years of age.

## Discussion

In 2012, the World Health Organization identified pregnant women and newborns as priority risk groups for seasonal influenza [8]. However, the full burden of influenza in pregnant women and their infants is poorly understood due to differences in the design and conduct of epidemiological studies, and the small numbers of pregnant women included [21–23]. The present study describes new epidemiological data of influenza infection among a large cohort of pregnant women, and the impact of influenza on clinical outcomes in these women and their infants. Using a prospective, active-surveillance study design as part of the GIHSN’s hospital-based surveillance [16], we collected data from more than 1700 pregnant women (825 with confirmed influenza) admitted to a hospital specializing in acute respiratory infections.

We restricted the analysis to women aged 15–44 years. A standard age range (often ages 16–49) is usually [24], but not always [25], chosen to define women of childbearing age. This age-range includes women who are less likely to be pregnant [24, 26], as was the case in our investigation – the number of pregnant women aged 45–50 was exceedingly small (< 1%, data not shown). In addition, the distribution of pregnant women in the lowest (15–19 year) and highest (40–44 year) age groups in our study was similar, and we restricted the childbearing age range (15 to < 44) according to pregnancy probability in our dataset to ensure consistency and reasonable estimates.

Influenza infection, confirmed by RT-PCR, accounted for nearly half of the admissions over the four influenza seasons. The risk of influenza infection was higher in pregnant than non-pregnant women (OR = 2.87 [95% CI, 2.10–3.92]), irrespective of subtype/lineage and trimester. This agrees with a larger multi-country study by the GIHSN during the 2012/2013 influenza season, which found an aOR of 3.84 (95% CI, 2.48–5.94) for influenza-related hospitalization in pregnant vs. non-pregnant women [16]. It also agrees with a meta-analysis of influenza A infection, which found a combined OR of 2.44 (95% CI 1.22–4.87) for influenza-related hospitalization in pregnant vs. non-pregnant women, although most of the included studies were during the 2009 A(H1N1) pandemic season [4].

Underlying conditions, especially anemia, obesity, and asthma, increase the risk of influenza-related hospitalization in pregnant women [27, 28]. Although we confirmed this in the present study, we were unable to detect significant differences for individual conditions, probably because of insufficient numbers. The present study also confirmed that the risk of hospitalization with influenza does not differ by trimester or influenza subtype, as described by others [29].

Influenza-positive pregnant admissions were hospitalized sooner after the onset of symptoms and stayed slightly longer in the hospital than influenza-negative pregnant admissions. Pregnant admissions positive for influenza also more frequently complained of cough, myalgia, and especially fever than those who were negative for influenza. This suggests that influenza causes more severe illness in pregnant women than other kinds of acute respiratory infection.

Influenza viruses varied substantially between seasons, although all subtypes and lineages resulted in hospitalization. In addition, demographics, clinical manifestations, and rates of stillbirth differed slightly between subtypes/lineages. For example, the risk of influenza infection increased slightly with the mother’s age. Also, admissions with influenza A(H1N1)pdm09 or B/Victoria lineage occurred mostly in the first or second trimester, whereas admissions with influenza A(H3N2) or B/Yamagata lineage occurred mostly in the second or third trimester. Although influenza B has been reported to be more frequent in the second trimester than influenza A [29], these results suggest that the two B lineages cannot be considered equivalent.

Influenza, especially A(H1N1), is considered a risk for stillbirth and low birth weight [27, 30–32]. However, we did not find differences in rates of stillbirth, preterm delivery, or caesarean delivery between influenza-positive and -negative pregnant admissions or between subtypes/lineages. In agreement with this, a recent meta-analysis reported a computed pooled OR of 1.24 (95% CI, 0.96–1.59) for small for gestational age, suggesting that influenza does not affect birth weight [23]. Unexpectedly, however, abortion before 20 weeks was more frequent in influenza-negative than influenza-positive women, suggesting that other respiratory infections pose a higher risk of abortion.

These data confirm that pregnant women are at increased risk from seasonal influenza A and B viruses. With more than 1700 pregnant admissions, this study provides important and detailed information about the impact of influenza in pregnant women that can be used to inform and support vaccination policies in this susceptible population. Furthermore, our study provides pregnancy outcome data, which are rarely included in epidemiological studies of influenza in pregnant women, and have not been published before.

However, this study had some limitations. The main analysis was based on comparing hospitalized influenza-positive with hospitalized influenza-negative pregnant women. We were unable to compare pregnant admissions to non-pregnant admissions because of insufficient numbers of non-pregnant women, who are more frequently admitted for influenza-like illness to other hospitals in Moscow. Nonetheless, the unique nature of the CHID#1 study site allowed us to recruit a substantial number of pregnant women. CHID#1 receives the most pregnant admissions from any of the hospitals in the GIHSN network (over 97% of the total pregnant admissions based on unpublished GIHSN data from the 2015/2016 season). Another limitation was that we could not assess the long-term effects of influenza on pregnant women, or pregnancy outcome beyond the current admission, because data were collected only from women while they were hospitalized, and follow-up evaluations until the end of pregnancy were not within the study protocol. Finally, the study could have been limited by increasing rates of hospitalization for pregnant women following the 2009 pandemic due to increased awareness of the risks. However, this was probably accounted for by recruiting consecutive admissions without previous knowledge of influenza status. Furthermore, influenza positivity rates were similar over the four influenza seasons, suggesting that this was not a problem.

## Conclusions

Our results confirm that pregnant women are at increased risk from influenza infection irrespective of season, circulating viruses, or trimester. This supports recommendations by the World Health Organization [8] and many countries [33, 34] that pregnant women be prioritized for seasonal influenza vaccination. Despite these recommendations and evidence that influenza vaccination is considered safe and effective for pregnant women [7, 10, 12, 35, 36], vaccination uptake by pregnant women is generally poor [37], as found in the present study, where < 1% of pregnant women were vaccinated. Additional efforts are therefore needed to educate healthcare workers, public health officials, and pregnant women about the risks of seasonal influenza and the importance of vaccination.

## Additional file

Additional file 1: Predicted probability of admission with influenza by (**a**) trimester, (**b-e**) subtype/lineage, and (**f**) overall according to age group and presence of underlying conditions. Conditional plots examining interactions between trimester and patient age or presence of underlying conditions for the risk of admission with any influenza or with each subtype/lineage. (PDF 35 kb)

## Abbreviations

aOR: Adjusted odds ratio
CHID#1: Federal Budget Institute of Health “Clinical Hospital for Infectious Diseases No. 1”
CI: Confidence interval
GIHSN: Global Influenza Hospital Surveillance Network
OR: Odds ratio
RT-PCR: Reverse transcription-polymerase chain reaction
