# Supplementary material for: Epidemiology of influenza in pregnant women hospitalized with respiratory illness in Moscow, 2012/2013–2015/2016: a hospital-based active surveillance study
Source: BMC Pregnancy Childbirth. 2019 Feb 15;19:72. doi: 10.1186/s12884-019-2192-z (PMC6377748; doi:10.1186/s12884-019-2192-z)
Supplement: Supplementary file 1 — Predicted probability of admission with influenza by (a) trimester, (b-e) subtype/lineage, and (f) overall according to age group and presence of underlying conditions. Conditional plots examining interactions between trimester and patient age or presence of underlying conditions for the risk of admission with any influenza or with each subtype/lineage. (PDF 35 kb) [file 12884_2019_2192_MOESM1_ESM.pdf]

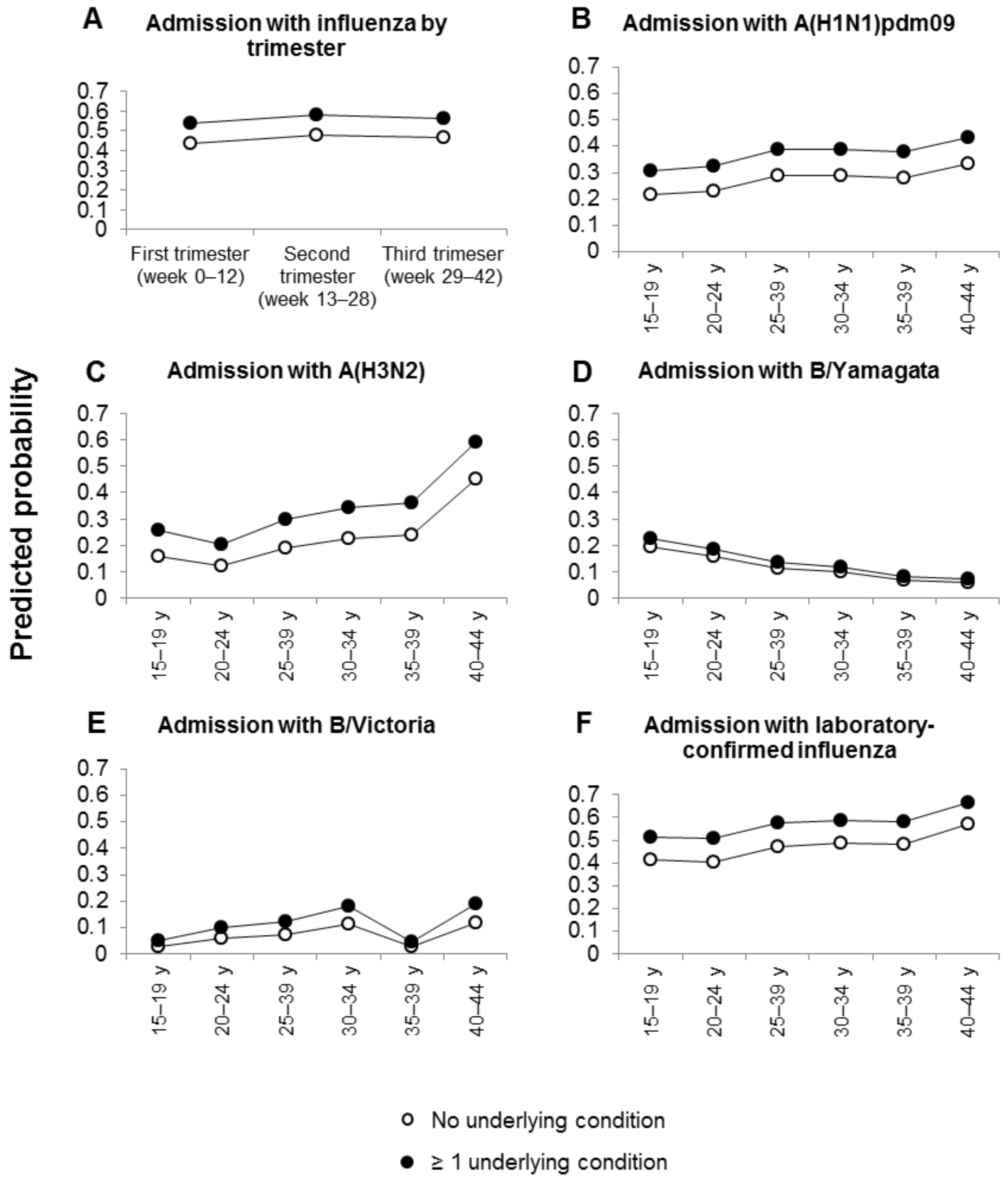

Predicted probability of admission with influenza by (A) trimester, (B-E) subtype/lineage, and (F) overall according to age group and presence of underlying conditions. Probabilities were adjusted by age (grouped in 5-year increments), smoking habits (yes/no), underlying conditions (yes/no), calendar time at admission (season-week) as splines (four knots), time from admission to swab in days (grouped), and admission during the previous 12 months (yes/no).
